# Association between maternal HBV-DNA levels and pregnancy outcomes among hepatitis B carriers: a retrospective cohort study in China

**DOI:** 10.1186/s12884-026-09040-1

**Published:** 2026-04-02

**Authors:** Xiumei Cai, Suiping Huang, Zuliang Ma

**Affiliations:** 1 Department of Medical Quality Control, Nanhai Affiliated Maternity and Children’s Hospital of Guangzhou University of Chinese Medicine, Foshan, Guangdong Province 528200 People’s Republic of China; 2 Department of Disease Prevention and Health Care, The Eighth Affiliated Hospital, Southern Medical University (The First People’s Hospital of Shunde, Foshan), Foshan, Guangdong Province 528308 People’s Republic of China

**Keywords:** Complications, HBV-DNA, Hepatitis B virus, Pregnancy outcomes

## Abstract

**Background:**

The relationship between maternal hepatitis B virus (HBV) DNA load and pregnancy outcomes remains inconsistent. Clarifying this association, including the potential dose-response relationship, is important for improving prenatal management.

**Methods:**

A retrospective study was conducted in a single-center cohort of 1,020 HBsAg-positive pregnant women. Participants were categorized by HBV-DNA load, and associations with pregnancy outcomes were assessed using multivariate logistic regression. Sensitivity analyses included multiple imputation for missing data and exclusion of women who received antiviral therapy during pregnancy.

**Results:**

The mean age was 32.51 ± 4.75 years, and the mean pre-pregnancy BMI was 21.43 ± 3.07 kg/m². Most participants were multiparous (67.65%) and had a gravidity ≥ 2 (77.16%). After full adjustment for covariates, women in the highest HBV-DNA load group (Group 3) had a higher risk of preterm birth than those in the lowest group (Group 1) (Odds ratio (OR): 5.36; 95% Confidence interval (CI): 2.43–11.82; *P* < 0.001). Median HBV-DNA levels were higher in the preterm birth group than in the non-preterm group. Restricted cubic spline analysis showed a positive association between maternal HBV-DNA load and preterm birth risk.

**Conclusions:**

This study found that high maternal HBV-DNA load was associated with an increased risk of preterm birth. These findings support the potential clinical value of prenatal HBV-DNA quantification in identifying pregnancies at high risk of preterm birth and enabling timely risk stratification and individualized management.

**Supplementary Information:**

The online version contains supplementary material available at 10.1186/s12884-026-09040-1.

## Introduction

Hepatitis B virus (HBV) is a partially double-stranded DNA virus that infects hepatocytes. HBV infection remains a major global public health issue. In 2022, approximately 254 million people were living with chronic hepatitis B infection [1]. The impact of HBV infection on maternal health during pregnancy and pregnancy outcomes has consistently attracted considerable attention. It is estimated that about 4.8% of pregnant women worldwide are infected with HBV [2].

Chronic HBV infection during pregnancy is associated with a persistent inflammatory state driven by ongoing viral replication, which may contribute to adverse pregnancy outcomes [3–6]. Previous studies have explored the relationship between maternal HBV infection and adverse pregnancy outcomes, however, many did not stratify HBV-DNA levels in detail and instead categorized them broadly as high versus low. In addition, important factors such as antiviral therapy during pregnancy and body mass index (BMI) have not been consistently considered. Moreover, findings on the impact of the HBV-DNA load on pregnancy outcomes remain inconsistent [7–9]. Limited evidence hinders the advancement of targeted monitoring and intervention strategies. Given these gaps, this study extends the existing literature by evaluating the dose-response relationship between maternal HBV-DNA levels and pregnancy outcomes, rather than merely confirming previously reported associations. We hypothesized that higher maternal HBV-DNA levels during pregnancy are associated with an increased risk of adverse pregnancy outcomes. Therefore, this retrospective single-center cohort study aimed to evaluate the association between maternal HBV-DNA load and pregnancy-related outcomes and to explore potential dose-response relationships.

## Materials and methods

### Study design and participants

**Fig. 1:**
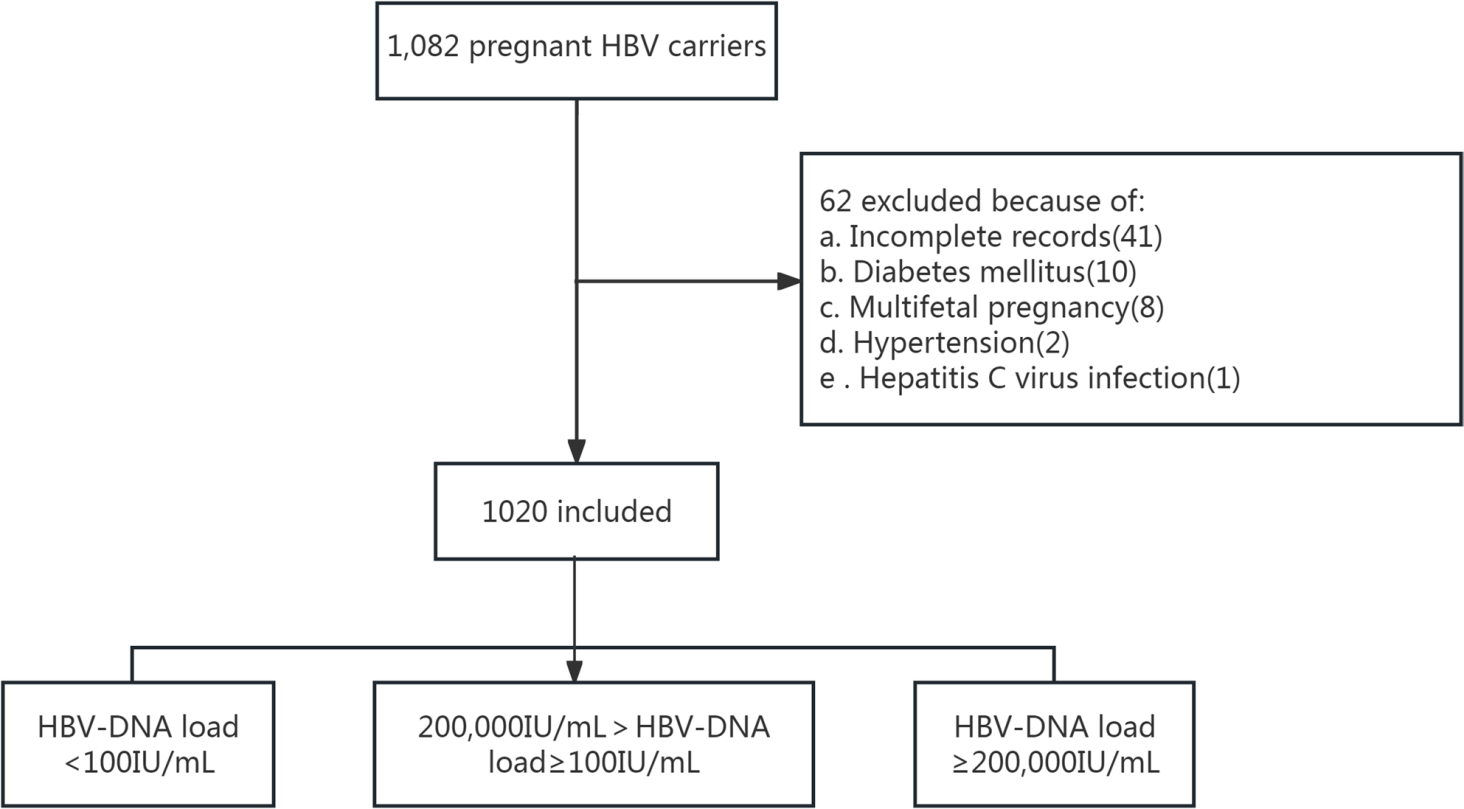
Flow chart for study population

We conducted a retrospective cohort study comprising pregnant women who tested positive for HBsAg and gave birth at The Eighth Affiliated Hospital of Southern Medical University between January 1, 2020, and December 31, 2022. Subjects were excluded from the study on the basis of the following criteria: (1) history of hypertension or diabetes; (2) coinfection with other hepatitis viruses, HIV, or syphilis; (3) age < 18 years; (4) multiple pregnancy; (5) severe liver pathology (such as cirrhosis or hepatocellular carcinoma); (6) concurrent diagnosis of malignant tumors or immunodeficiency disorders; (7) incomplete information. Following a comprehensive screening of 1,082 pregnancies, 62 participants were excluded based on the predefined criteria, yielding a final study cohort of 1,020 pregnant women (Fig. 1).

All pregnant women were screened for hepatitis B surface antigen (HBsAg) at their booking appointment, and those who tested positive subsequently underwent HBV-DNA testing. Among the participants, 351 (34.41%) underwent testing during early pregnancy, whereas 669 (65.59%) were tested during mid-pregnancy. All HBV-DNA measurements were performed using fluorescence quantitative polymerase chain reaction (FQ-PCR) methodology with a quantifiable range of 10² to 5 × 10^8^ IU/mL. Based on HBV-DNA levels, participants were stratified into three groups: Group 1 (< 10² IU/mL), Group 2 (10²-2 × 10⁵ IU/mL), and Group 3 (≥ 2 × 10⁵ IU/mL). These cut-off values were defined according to assay detection limits and established clinical thresholds for antiviral intervention during pregnancy [10].

### Outcome measures

All the following data were collected from the hospital’s electronic medical record system: (1) maternal characteristics such as age, gravidity, parity, pre-pregnancy BMI, method of delivery, profession, residential status, marital status, ethnicity, method of conception, history of cesarean delivery, history of preterm birth, and history of spontaneous abortion; (2) maternal complications included gestational hypertension, postpartum hemorrhage (PPH), gestational diabetes mellitus (GDM), Intrahepatic cholestasis of pregnancy (ICP), premature rupture of membrane (PROM), anemia during pregnancy; (3) neonatal outcomes: gestational age (weeks); (4) laboratory investigations: HBV-DNA, alanine aminotransferase (ALT).

### Diagnostic criteria

The diagnosis of GDM was established in accordance with the guidelines of the National Health Commission of the People’s Republic of China. Accordingly, all pregnant women without a prior diagnosis of diabetes were universally screened with a 75 g Oral Glucose Tolerance Test (OGTT) at 24–28 weeks of gestation. GDM was diagnosed if any one of the following plasma glucose thresholds was met or exceeded: fasting ≥ 5.1 mmol/L, 1-hour ≥ 10.0 mmol/L, or 2-hour ≥ 8.5 mmol/L. Gestational hypertension was defined as a sustained elevation in blood pressure, documented on at least two separate occasions at least 4 h apart, characterized by a systolic blood pressure (SBP) ≥ 140 mmHg and/or a diastolic blood pressure (DBP) ≥ 90 mmHg, emerging after the 20th week of gestation in women with no history of hypertension. According to the 2015 guideline of the Chinese Medical Association, ICP was defined as fasting serum TBA concentrations ≥ 10 µmol/L in the absence of other causes of pruritus or liver dysfunction [11]. PPH was defined according to standard obstetric definitions as a blood loss of ≥ 500 mL following vaginal delivery or ≥ 1000 mL during cesarean section, occurring within the first 24 h after birth. Preterm birth was characterized by birth occurring before the completion of 37 weeks of gestation.

### Statistical analyses

All analyses were performed using R software (version 4.2.2) and Free Statistics analysis platform (version 2.2). Continuous variables were summarized as mean ± standard deviation (SD) for normally distributed data and as median (interquartile range (IQR)) for non-normally distributed data. Between-group differences were evaluated using one-way analysis of variance (ANOVA), the Mann-Whitney U test, or the Kruskal-Wallis test, as appropriate, implemented in R (*stats* package). Categorical variables were expressed as n (%), and were analyzed by the χ² test using the *stats* package. HBV-DNA load was log10-transformed for analysis. Samples with results below the lower limit of quantification (< 10^2^ IU/mL) were assigned a value of 50 IU/mL, while those exceeding the upper limit (> 5 × 10^8^ IU/mL) were assigned 5 × 10^8^ IU/mL for statistical comparisons. Multivariable logistic regression models were constructed using the *stats* package. Results are reported as odds ratio (OR) with corresponding 95% confidence interval (CI). The group with the lowest HBV-DNA load was selected as the reference group. In model I, we adjusted for the factors that differed significantly among three groups, including age, profession, ethnicity, antiviral therapy, and ALT. In model II, we additionally adjusted for pre-pregnancy BMI, gravidity, parity, residential status, marital status, in vitro fertilization (IVF), anemia during pregnancy, GDM, history of cesarean delivery, history of preterm birth, and history of spontaneous abortion. Nonlinear associations were evaluated using restricted cubic splines (RCS) implemented with the *rms* package (knots at the 5th, 35th, 65th, and 95th percentiles). Sensitivity analyses were conducted using the imputed datasets and by excluding women who received antiviral therapy during pregnancy to assess the robustness of the findings. Missing data were handled using multiple imputation by chained equations (MICE) implemented in the *mice* package. Under the missing at random (MAR) assumption, 5 imputed datasets were generated. The imputation model included all variables in Model II of the multivariable logistic regression analysis, as well as the log-transformed HBV-DNA level. Data visualization was performed using the *ggplot2* package. *P*-values < 0.05 were considered statistically significant.

### Reporting guideline

This study was prepared and presented in line with the Strengthening the Reporting of Observational Studies in Epidemiology (STROBE) statement for cohort studies [12].

## Results

### Baseline characteristics

Significant differences were observed among the three groups in age, profession, ethnicity, antiviral therapy, and ALT, whereas the other baseline characteristics were comparable (Table 1).

**Table 1:** Baseline characteristics of the study participants

	Total (*n* = 1020)	Group 1 (*n* = 533)	Group 2 (*n* = 330)	Group 3 (*n* = 157)	*P*
Age (years)	32.51 ± 4.75	32.53 ± 4.52	32.97 ± 4.70	31.48 ± 5.46	0.005
Pre-pregnancy BMI(kg/m^2^)	21.43 ± 3.07	21.39 ± 3.09	21.47 ± 3.03	21.51 ± 3.15	0.889
Gravidity					0.441
1 (*n*%)	233 (22.84)	119 (22.33)	72 (21.82)	42 (26.75)	
≥ 2 (*n*%)	787 (77.16)	414 (77.67)	258 (78.18)	115 (73.25)	
Parity					0.484
1 (*n*%)	330 (32.35)	171 (32.08)	102 (30.91)	57 (36.31)	
≥ 2 (*n*%)	690 (67.65)	362 (67.92)	228 (69.09)	100 (63.69)	
Profession					0.009
Employee (*n*%)	593 (58.14)	309 (57.97)	208 (63.03)	76 (48.41)	
Housewife/unemployed (*n*%)	427 (41.86)	224 (42.03)	122 (36.97)	81 (51.59)	
Residential status					0.404
Permanent residents (*n*%)	535 (52.45)	289 (54.22)	170 (51.52)	76 (48.41)	
Temporary residents (*n*%)	485 (47.55)	244 (45.78)	160 (48.48)	81 (51.59)	
Marital status					0.258
Married (*n*%)	1006 (98.63)	528 (99.06)	325 (98.48)	153 (97.45)	
Unmarried/Divorced (*n*%)	14 ( 1.37)	5 (0.94)	5 (1.52)	4 (2.55)	
IVF					0.155
No (*n*%)	988 (96.86)	511 (95.87)	324 (98.18)	153 (97.45)	
Yes (*n*%)	32 ( 3.14)	22 (4.13)	6 (1.82)	4 (2.55)	
Anemia during pregnancy					0.377
No (*n*%)	730 (71.57)	381 (71.48)	230 (69.70)	119 (75.80)	
Yes (*n*%)	290 (28.43)	152 (28.52)	100 (30.30)	38 (24.20)	
Ethnicity					0.017
Others (*n*%)	14 ( 1.37)	7 (1.31)	1 (0.30)	6 (3.82)	
Han (*n*%)	1006 (98.63)	526 (98.69)	329 (99.70)	151 (96.18)	
History of cesarean section					0.224
No (*n*%)	778 (76.27)	399 (74.86)	251 (76.06)	128 (81.53)	
Yes (*n*%)	242 (23.73)	134 (25.14)	79 (23.94)	29 (18.47)	
History of preterm birth					0.745
No (*n*%)	994 (97.45)	519 (97.37)	323 (97.88)	152 (96.82)	
Yes (*n*%)	26 ( 2.55)	14 (2.63)	7 (2.12)	5 (3.18)	
History of spontaneous abortion					0.791
No (*n*%)	888 (87.06)	461 (86.49)	288 (87.27)	139 (88.54)	
Yes *n*%)	132 (12.94)	72 (13.51)	42 (12.73)	18 (11.46)	
Antiviral therapy					< 0.001
No (*n*%)	763 (74.80)	439 (82.36)	280 (84.85)	44 (28.03)	
Yes (*n*%)	257 (25.20)	94 (17.64)	50 (15.15)	113 (71.97)	
ALT (U/L)	16.00 (12.00, 24.00)	15.00 (11.00, 20.00)	16.00 (12.00, 22.00)	24.00 (16.00, 38.00)	< 0.001

Abbreviations: *BMI* body mass index, *IVF* in vitro fertilization, *ALT* alanine aminotransferase

### Correlation between HBV-DNA levels and pregnancy outcomes

**Fig. 2:**
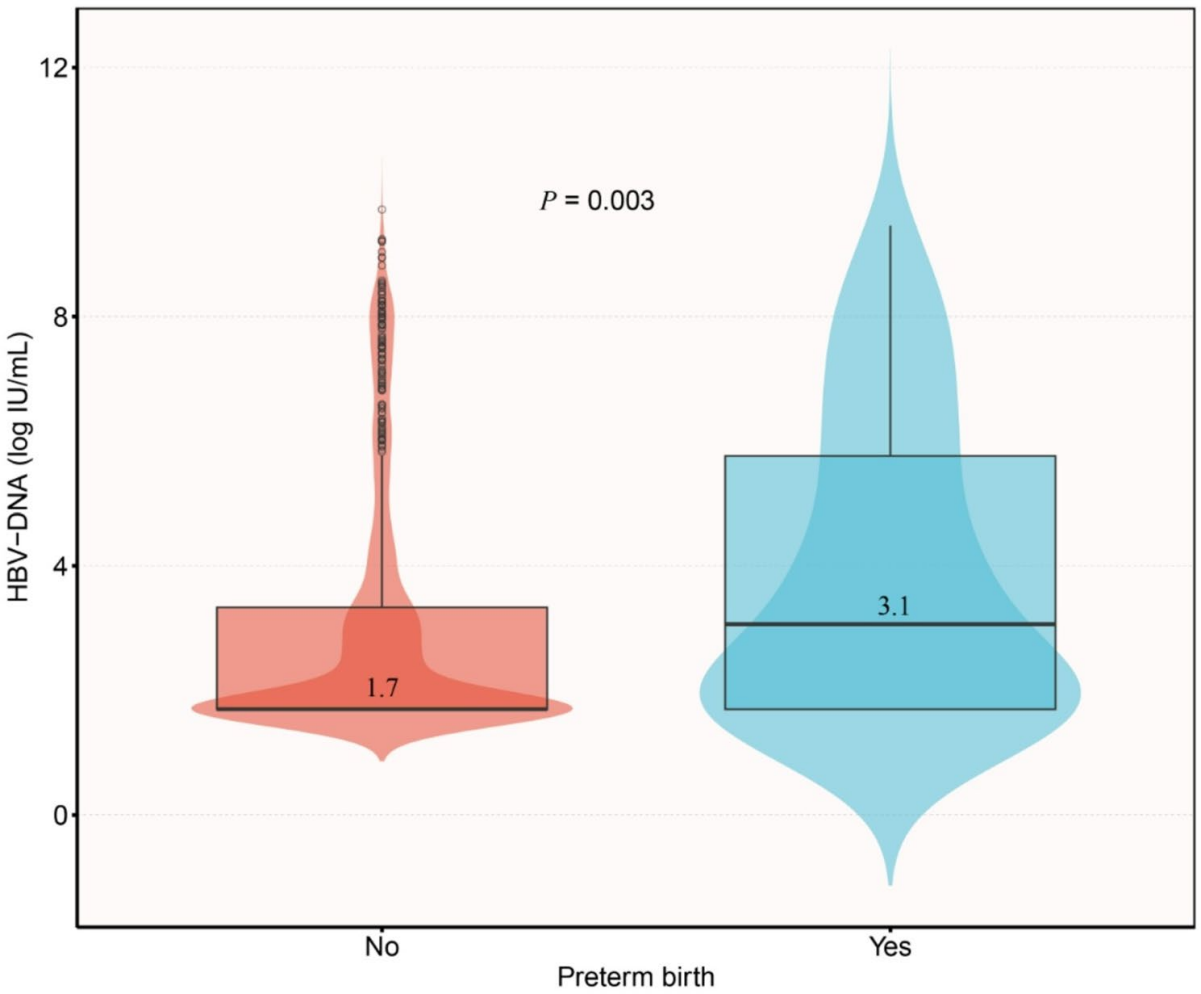
Comparison of HBV-DNA load between preterm birth and non-preterm birth groups. Notes: Violin plots with overlaid boxplots illustrate the distribution of HBV-DNA levels (log IU/mL) in the two groups

As shown in Table 2, after full adjustment for covariates in Model II, participants in the highest HBV-DNA load group (Group 3) had a significantly increased risk of preterm birth compared with those in the lowest group (Group 1). Consistently, Fig. 2 shows that HBV-DNA levels were significantly higher in the preterm birth group than in the non-preterm birth group.

**Table 2:** Pregnancy outcomes with respect to HBV-DNA levels

Variable	Total (*N*)	Event (*n*(%))	Unadjusted Model		Model I^*^		Model II^†^		
			crude.OR (95%CI)	crude.*P*	adj.OR (95%CI)	adj.*P*	adj.OR (95%CI)		adj.*P*
Preterm birth									
Group 1	533	26 (4.88)	1.00 (Ref)		1.00 (Ref)		1.00 (Ref)		
Group 2	330	20 (6.06)	1.26 (0.69–2.29)	0.453	1.23 (0.67–2.26)	0.500	1.27 (0.69–2.37)		0.443
Group 3	157	18 (11.46)	2.53 (1.35–4.74)	0.004	5.99 (2.81–12.79)	< 0.001	5.36 (2.43–11.82)		< 0.001
Trend.test				0.007		< 0.001			< 0.001
Gestational hypertension									
Group 1	533	6 (1.13)	1.00 (Ref)		1.00 (Ref)		1.00 (Ref)		
Group 2	330	3 (0.91)	0.81 (0.20–3.24)	0.761	0.71 (0.17–2.90)	0.633	0.84 (0.19–3.73)		0.818
Group 3	157	1 (0.64)	0.56 (0.07–4.71)	0.596	1.39 (0.14–14.31)	0.78	1.66 (0.13–21.72)		0.699
Trend.test				0.572		0.903			0.919
GDM									
Group 1	533	117 (21.95)	1.00 (Ref)		1.00 (Ref)		1.00 (Ref)		
Group 2	330	69 (20.91)	0.94 (0.67–1.31)	0.718	0.91 (0.65–1.29)	0.595	0.92 (0.65–1.31)		0.65
Group 3	157	35 (22.29)	1.02 (0.66–1.57)	0.928	1.58 (0.95–2.65)	0.08	1.54 (0.9–2.62)		0.115
Trend.test				0.956		0.292			0.339
PROM									
Group 1	533	117 (21.95)	1.00 (Ref)		1.00 (Ref)		1.00 (Ref)		
Group 2	330	58 (17.58)	0.76 (0.53–1.08)	0.121	0.74 (0.52–1.06)	0.1	0.7 (0.49-1)		0.052
Group 3	157	35 (22.29)	1.02 (0.66–1.57)	0.928	1.23 (0.75-2)	0.417	1.22 (0.74–2.01)		0.446
Trend.test				0.629		0.878			0.734
ICP									
Group 1	533	13 (2.44)	1.00 (Ref)		1.00 (Ref)		1.00 (Ref)		
Group 2	330	3 (0.91)	0.37 (0.1–1.3)	0.12	0.37 (0.11–1.32)	0.126	0.33 (0.09–1.21)		0.095
Group 3	157	1 (0.64)	0.26 (0.03–1.97)	0.191	0.26 (0.03–2.21)	0.217	0.24 (0.03–2.06)		0.194
Trend.test				0.068		0.071			0.055
Postpartum hemorrhage									
Group 1	533	23 (4.32)	1.00 (Ref)		1.00 (Ref)		1.00 (Ref)		
Group 2	330	10 (3.03)	0.69 (0.33–1.47)	0.341	0.69 (0.33–1.49)	0.348	0.65 (0.3–1.42)		0.283
Group 3	157	9 (5.73)	1.35 (0.61–2.98)	0.459	1.49 (0.59–3.77)	0.397	1.31 (0.51–3.35)		0.578
Trend.test				0.751		0.789			0.996

^*^Model I adjustment covariates are age, profession, ethnicity, antiviral therapy, and ALT

^†^Model II adjustment covariates are age, profession, ethnicity, antiviral therapy, ALT, pre-pregnancy BMI, gravidity, parity, residential status, marital status, in vitro fertilization (IVF), anemia during pregnancy, GDM, history of cesarean delivery, history of preterm birth, and history of spontaneous abortion

Abbreviations: *GDM* gestational diabetes mellitus, *PROM* premature rupture of membrane, *ICP* intrahepatic cholestasis of pregnancy, *ALT* alanine aminotransferase

### Dose-response relationship between the HBV-DNA levels and preterm birth

**Fig. 3:**
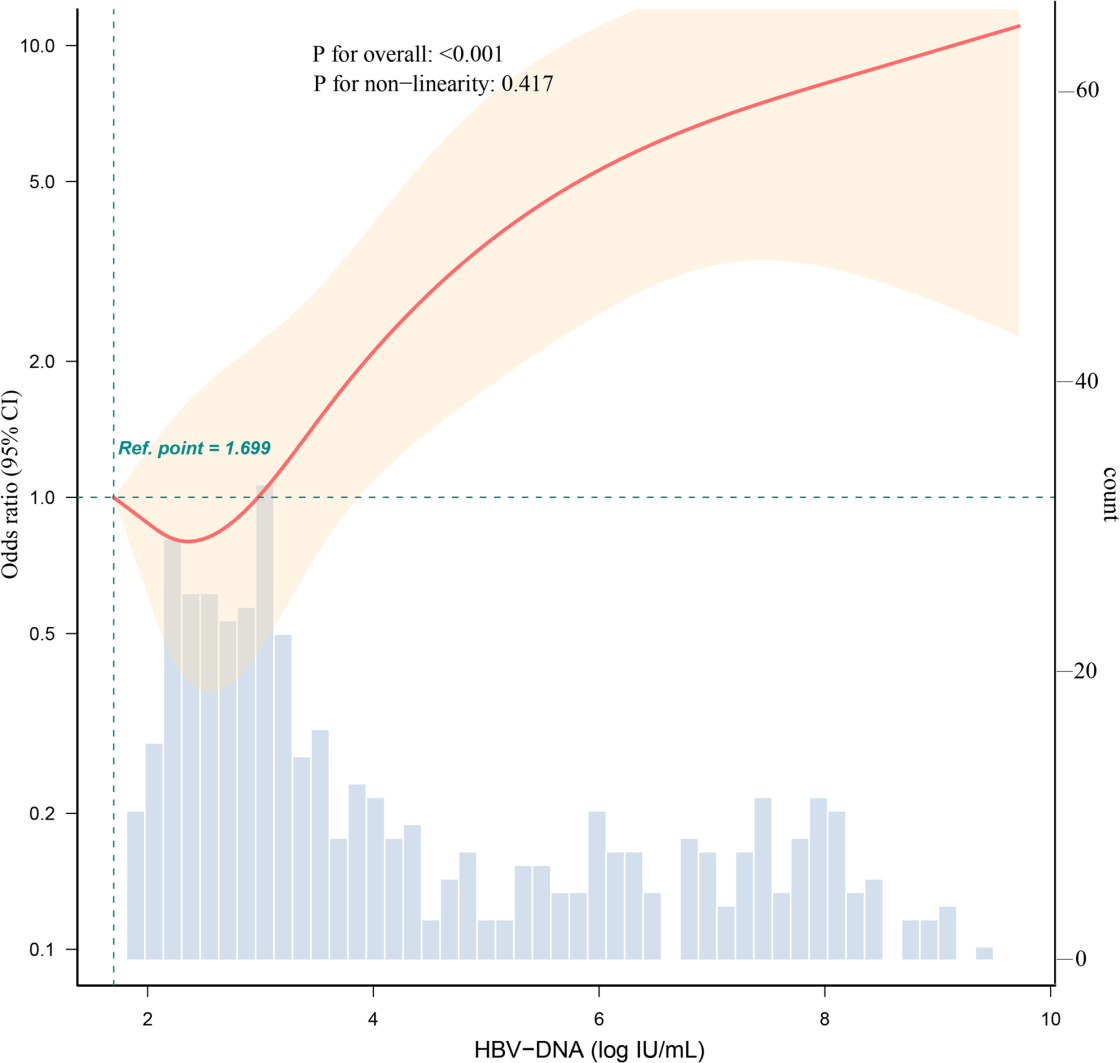
Association between preterm birth and HBV-DNA load by RCS. Notes: Dashed vertical line represent reference point of 1.699 log IU/mL (≈50 IU/mL). Dashed horizontal line depict an OR of 1.0. Red line represent the estimated OR, while shaded ribbon depict a 95% CI. The model was adjusted according to Model II

RCS analysis showed a positive association between maternal HBV-DNA levels and preterm birth risk (Fig. 3). The overall association was statistically significant, with no evidence of nonlinearity, suggesting an approximately linear increase in risk with rising maternal HBV-DNA levels.

### Sensitivity analysis

Sensitivity analysis confirmed the robustness of the findings. A total of 41 (4.02%) participants had missing data. After multiple imputation, the highest HBV-DNA group (Group 3) remained significantly associated with an increased risk of preterm birth compared with the lowest group (Group 1). After excluding women who received antiviral therapy, the association persisted with a comparable magnitude. These results suggest that the association between higher HBV-DNA levels and preterm birth was not materially affected by missing data or antiviral treatment (Additional file: Table A2–A3 and Fig. A1–A4).

## Discussion

This study provides evidence on the association between maternal HBV-DNA load and adverse perinatal outcomes. Notably, maternal HBV-DNA load was associated with an increased risk of preterm birth. These findings suggest that a high HBV-DNA load is not merely a marker of infectiousness, but may also be associated with specific adverse neonatal outcomes.

Preterm birth not only significantly contributes to morbidity and mortality in children globally but also elevates the risk of mortality from infancy to adulthood [13]. Therefore, identifying risk factors for preterm birth is crucial. Although numerous studies have explored the relationship between maternal HBV-DNA load and preterm birth, the findings remain inconsistent. Several studies reported no association [7, 9, 14, 15], whereas others suggested that higher maternal HBV-DNA levels are associated with an increased risk of preterm birth [8, 16, 17]. For example, one study reported a modest increase in risk (OR: 1.18) at a relatively low threshold of 1,000 copies/mL [17]. In contrast, our study observed a substantially stronger association (OR: 5.93) at a higher threshold of 2 × 10^5^ IU/mL. These inconsistencies may be partly explained by differences in exposure definition and risk stratification. For instance, a study dichotomized maternal HBV-DNA load as “low” (≤ 10^3^ IU/mL) versus “high” (> 10^3^ IU/mL) and reported no statistically significant association with preterm birth [7]. Such a cut-off may be insufficient to distinguish women with truly high-level viremia from those with only modest viral replication, thereby diluting the observable effect. In addition, differences in sample size, HBV genotype distribution, and adjustments for confounding factors may further contribute to the heterogeneity observed across studies.

The mechanism underlying the association between HBV-DNA and preterm birth may be as follows. Excessive production of proinflammatory cytokines significantly contributes to premature labor by causing inflammation [18–20]. Active HBV replication can induce a state of chronic systemic inflammation, characterized by elevated circulating proinflammatory cytokines [21]. This inflammatory milieu may predispose to placental dysfunction and trigger proinflammatory cascades that disrupt pregnancy maintenance, ultimately culminating in preterm labor [22–24]. In addition, the liver plays a vital role in modulating local and systemic inflammation [25]. Sustained HBV replication may also cause hepatic damage, thereby impairing the liver’s capacity to regulate inflammation.

In clinical practice, HBV-DNA quantification is widely used to stratify mother-to-child transmission (MTCT) risk and guide antiviral therapy during pregnancy in women with high HBV-DNA load, with the aim of reducing maternal HBV-DNA levels at delivery. In this context, our findings suggest that maternal HBV-DNA monitoring may also have broader clinical value in identifying HBV-positive women at increased risk of preterm birth. Maternal HBV-DNA monitoring may help stratify HBV-positive pregnancies according to preterm birth risk. Incorporating quantitative HBV-DNA measurement into prenatal care may therefore contribute not only to MTCT prevention, but also to more individualized management for women with high HBV-DNA load. For these women, a tailored management strategy may be warranted, including intensified antenatal surveillance and, when clinically indicated, timely initiation of antiviral therapy. In addition, preterm delivery may complicate the implementation of perinatal prevention strategies by shortening the time window available for antenatal management. However, it is important to emphasize that MTCT outcomes were not assessed in the present study (e.g., infant HBsAg or HBV-DNA were not collected), and thus we cannot determine whether preterm birth in our cohort translated into higher MTCT risk. Given the substantial morbidity associated with prematurity, particularly among early preterm infants [26, 27], future mother-infant paired studies with detailed data on infant infection outcomes are warranted to clarify the interplay between maternal HBV-DNA load, preterm birth and MTCT prevention.

Beyond its established role in assessing MTCT risk, maternal HBV-DNA load reflects viral replication activity and may be relevant to a broader range of pregnancy outcomes. Regarding maternal complications, some studies have suggested associations with ICP and PPH [7, 28], whereas others reported no significant associations with outcomes such as GDM, gestational hypertension, PROM, and pre-eclampsia [7, 9, 17]. Consistent with these negative findings, our study did not observe significant associations between HBV-DNA load and maternal complications. For neonatal outcomes, prior evidence indicates that HBV-DNA load may be associated with neonatal asphyxia [7], while growth-related outcomes, including low birth weight and small for gestational age (SGA), are generally not associated with HBV-DNA load [7, 9, 17]. These mixed findings may be explained by differences in HBV-DNA cut-off values, antiviral therapy exposure, and confounder adjustment strategies.

This study has several limitations that should be acknowledged. First, the retrospective cohort design limits the ability to establish a causal relationship between maternal HBV-DNA levels and preterm birth. In addition, due to the small number of events in certain subgroups, some estimates had wide confidence intervals, and these results should therefore be interpreted with caution. Second, HBV-DNA was measured at varying gestational time points rather than a standardized time point, although prior evidence suggests that maternal HBV-DNA levels are relatively stable during pregnancy [29]. Third, although we adjusted for several important maternal and obstetric factors, residual confounding cannot be completely excluded, as is common in observational studies. Several potentially important variables were not available in our study. These include behavioral factors such as smoking, alcohol consumption, maternal nutritional status, antenatal care adherence, and lifestyle-related stress [30–33]. In addition, socioeconomic and occupational characteristics, including occupation type and prolonged standing during work, may also influence the risk of preterm birth but were not captured in this study [34]. Fourth, Although routine screening for syphilis and HIV was performed, other maternal infections and obstetric conditions known to increase the risk of preterm birth were not systematically evaluated, such as cervical insufficiency, Neisseria gonorrhoeae [35]. Finally, although age was adjusted for in the multivariable models, residual confounding related to advanced age cannot be entirely excluded. Therefore, future multicenter prospective studies are warranted to incorporate standardized data collection and to confirm our results.

## Conclusions

In conclusion, this study suggests that a high maternal HBV-DNA load may be associated with an increased risk of preterm birth. These findings may contribute to improved risk stratification and individualized management among HBsAg-positive pregnant women. However, given the retrospective single-center design, these results should be interpreted with caution. Further large-scale multicenter prospective studies with standardized data collection are warranted to validate these findings and to clarify their clinical implications.

## Supplementary Information

Additional file 1: Fig. A1 Comparison of HBV-DNAload between preterm and non-preterm birth groups after multiple imputation; Fig. A2 Association between preterm birth and HBV-DNA load by RCS after multiple imputation; Fig. A3 Comparison of HBV-DNA load between preterm and non-preterm birth groups after exclusion of women who received antiviral therapy; Fig. A4 Association between preterm birth and HBV-DNA load by RCS after exclusion of women who received antiviral therapy.

Additional file 2: Table A1. STROBE 2007 (v4) Statement—Checklist of items of cohort studies; Table A2. Association between preterm birth and HBV-DNA load after multiple imputation; Table A3. Association between preterm birth and HBV-DNA load after exclusion of women who received antiviral therapy.

## Data Availability

The datasets supporting the conclusions of this article are included within the article and its additional files. Further inquiries can be directed to the corresponding author.

## Abbreviations

BMI: Body mass index
IVF: In vitro fertilization
GDM: Gestational diabetes mellitus
PROM: Premature rupture of membrane
ICP: Intrahepatic cholestasis of pregnancy
HBeAg: Hepatitis B e antigen
HBV: Hepatitis B virus
HBsAg: Hepatitis B surface antigen
OGTT: Oral Glucose Tolerance Test
WHO: World Health Organization
CI: Confidence interval
OR: Odds ratio
ALT: Alanine aminotransferase
MTCT: Mother-to-child transmission
PPH: Postpartum hemorrhage
SGA: Small for gestational age
SBP: Systolic blood pressure
DBP: Diastolic blood pressure
ANOVA: Analysis of variance
