# Supplementary material for: Association between maternal HBV-DNA levels and pregnancy outcomes among hepatitis B carriers: a retrospective cohort study in China
Source: BMC Pregnancy Childbirth. 2026 Apr 2;26:521. doi: 10.1186/s12884-026-09040-1 (PMC13169610; doi:10.1186/s12884-026-09040-1)
Supplement: Supplementary file 1 — Additional file 1: Fig. A1 Comparison of HBV-DNAload between preterm and non-preterm birth groups after multiple imputation; Fig. A2 Association between preterm birth and HBV-DNA load by RCS after multiple imputation; Fig. A3 Comparison of HBV-DNA load between preterm and non-preterm birth groups after exclusion of women who received antiviral therapy; Fig. A4 Association between preterm birth and HBV-DNA load by RCS after exclusion of women who received antiviral therapy. [file 12884_2026_9040_MOESM1_ESM.pdf]

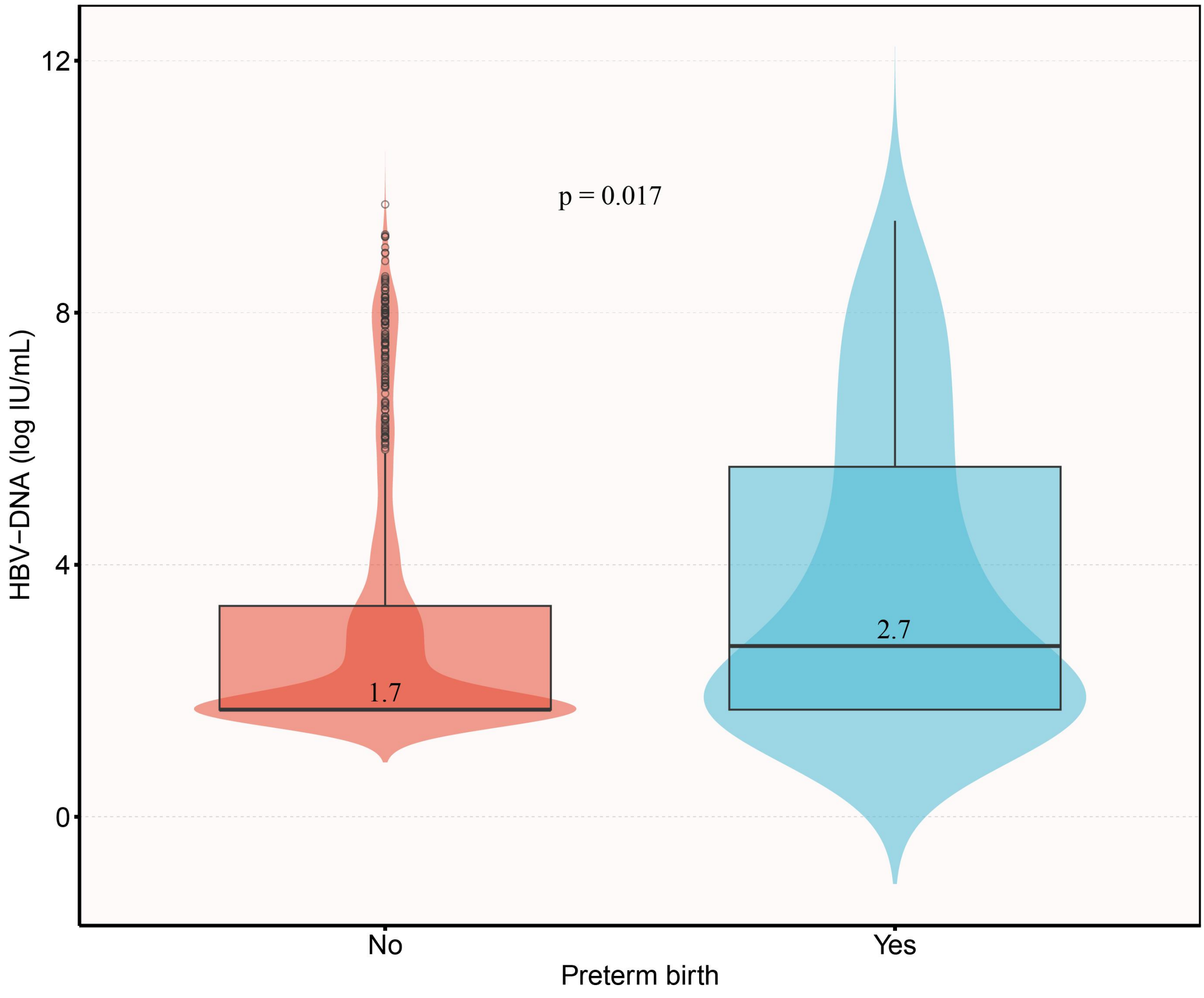

Additional file Fig. A1 Comparison of HBV-DNA load between preterm and non-preterm birth groups after multiple imputation.

Notes: Violin plots with overlaid boxplots illustrate the distribution of log10-transformed HBV-DNA levels (IU/mL) among women with and without preterm birth. The central line within each box represents the median, and the box indicates the interquartile range (IQR). The width of each violin reflects the density of observations. Data were derived from pooled datasets after multiple imputation for missing values.

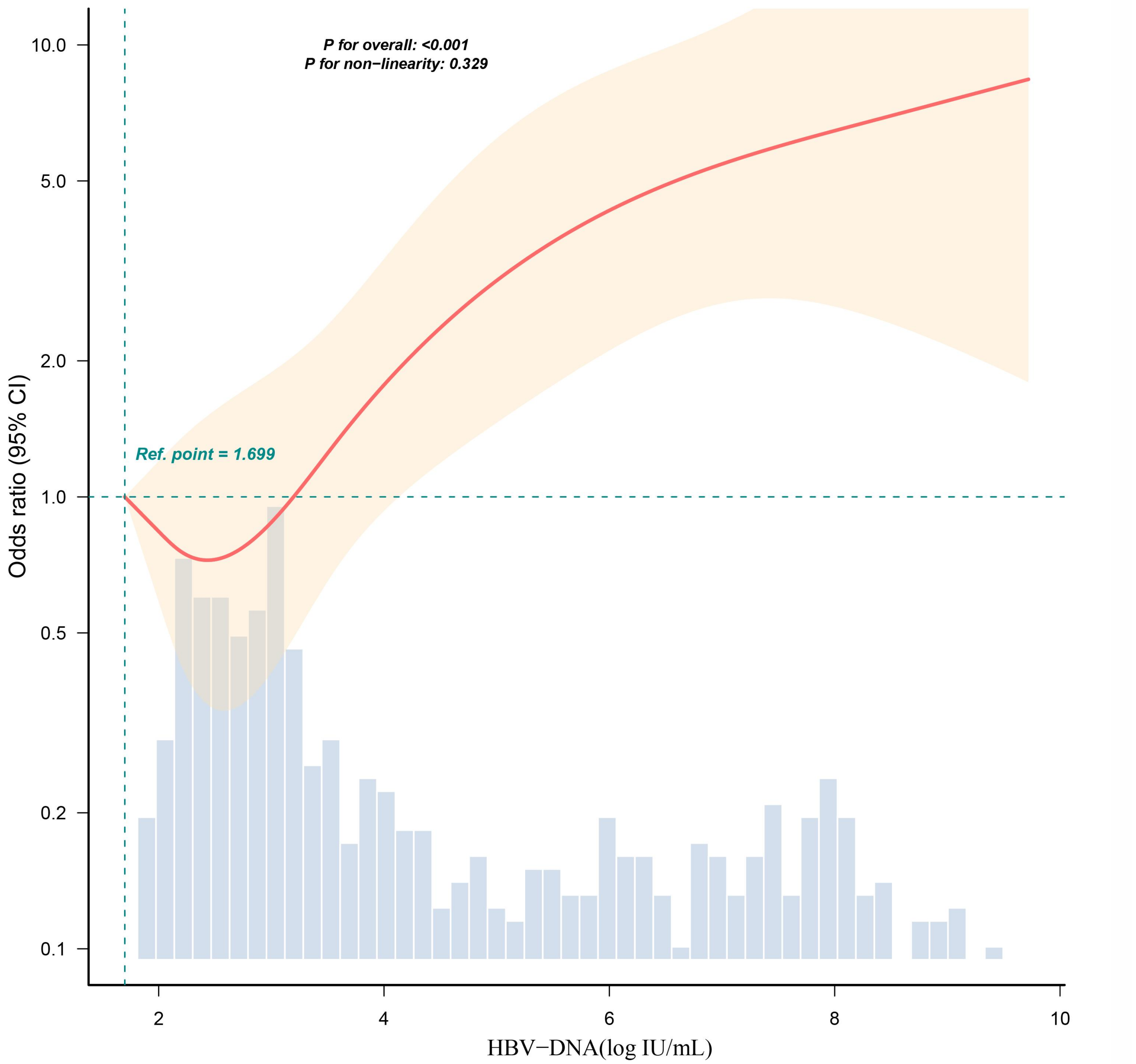

Additional file Fig. A2 Association between preterm birth and HBV-DNA load by RCS after multiple imputation.

Notes: Dashed vertical line represent reference point of 1.699 log IU/mL (≈50 IU/mL). Dashed horizontal line depict an OR of 1.0. Red line represent the estimated OR, while shaded ribbon depict a 95% CI. The model was adjusted according to Model II.

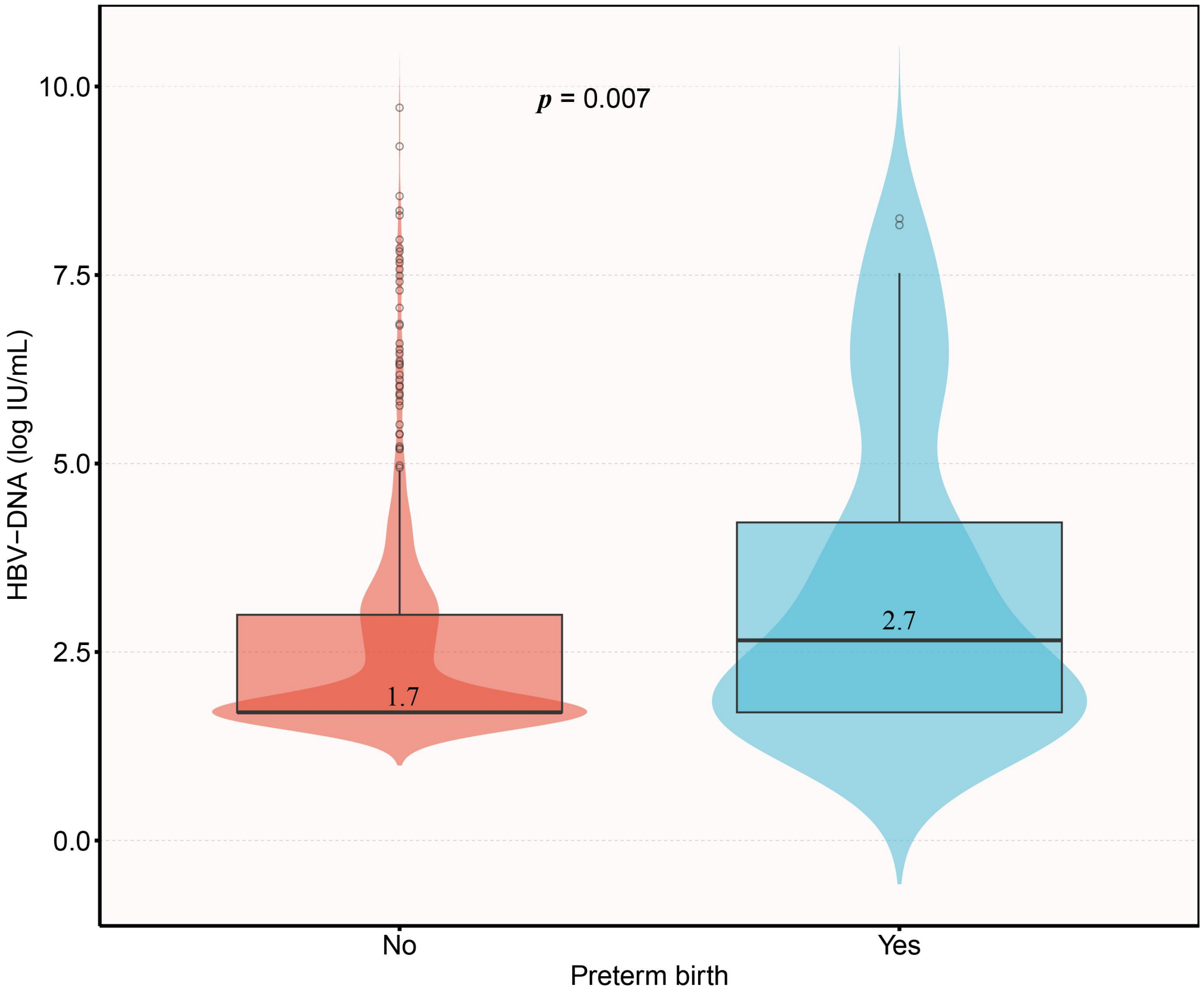

Additional file Fig. A3 Comparison of HBV-DNA load between preterm and non-preterm birth groups after exclusion of women who received antiviral therapy.

Notes: Violin plots with overlaid boxplots illustrate the distribution of log10-transformed HBV-DNA levels (IU/mL) among women with and without preterm birth. The central line within each box represents the median, and the box indicates the interquartile range (IQR). The width of each violin reflects the density of observations. Data were derived from pooled datasets after multiple imputation for missing values.

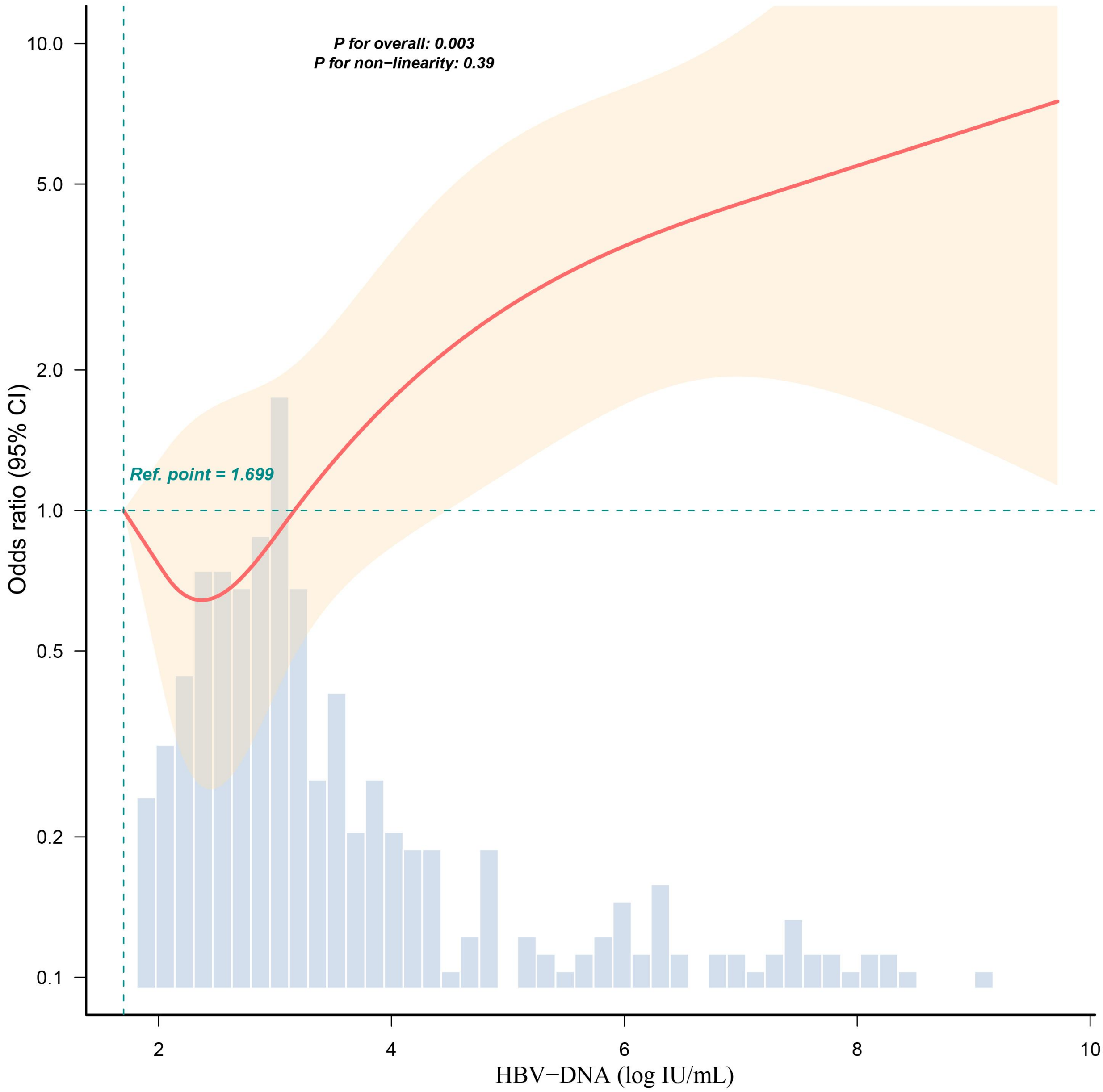

Additional file Fig. A4 Association between preterm birth and HBV-DNA load by RCS after exclusion of women who received antiviral therapy.

Notes: Dashed vertical line represent reference point of 1.699 log IU/mL (≈50 IU/mL). Dashed horizontal line depict an OR of 1.0. Red line represent the estimated OR, while shaded ribbon depict a 95% CI. The model was adjusted according to Model II.
