# Supplementary material for: Association between maternal HBV-DNA levels and pregnancy outcomes among hepatitis B carriers: a retrospective cohort study in China
Source: BMC Pregnancy Childbirth. 2026 Apr 2;26:521. doi: 10.1186/s12884-026-09040-1 (PMC13169610; doi:10.1186/s12884-026-09040-1)
Supplement: Supplementary file 2 — Additional file 2: Table A1. STROBE 2007 (v4) Statement—Checklist of items of cohort studies; Table A2. Association between preterm birth and HBV-DNA load after multiple imputation; Table A3. Association between preterm birth and HBV-DNA load after exclusion of women who received antiviral therapy. [file 12884_2026_9040_MOESM2_ESM.docx]

****Additional file Table A1.**** STROBE 2007 (v4) Statement—Checklist of items of cohort studies.

| **Section/Topic** | Recommendation | Reported on page # |
| --- | --- | --- |
| **Title and abstract** | (*a*) Indicate the study’s design with a commonly used term in the title or the abstract | 1 |
|  | (*b*) Provide in the abstract an informative and balanced summary of what was done and what was found | 1,2 |
|  |  |  |
| Background/rationale | Explain the scientific background and rationale for the investigation being reported | 2 |
| Objectives | State specific objectives, including any prespecified hypotheses | 2 |
|  |  |  |
| Study design | Present key elements of study design early in the paper | 3 |
| Setting | Describe the setting, locations, and relevant dates, including periods of recruitment, exposure, follow-up, and data collection | 3 |
| Participants | (*a*) Give the eligibility criteria, and the sources and methods of selection of participants. Describe methods of follow-up | 3 |
|  | (*b*) For matched studies, give matching criteria and number of exposed and unexposed | N/A |
| Variables | Clearly define all outcomes, exposures, predictors, potential confounders, and effect modifiers. Give diagnostic criteria, if applicable | 4 |
| Data sources/ measurement | For each variable of interest, give sources of data and details of methods of assessment (measurement). Describe comparability of assessment methods if there is more than one group | 4,5 |
| Bias | Describe any efforts to address potential sources of bias | 5 |
| Study size | Explain how the study size was arrived at | 3 |
| Quantitative variables | Explain how quantitative variables were handled in the analyses. If applicable, describe which groupings were chosen and why | 5 |
| Statistical methods | (*a*) Describe all statistical methods, including those used to control for confounding | 5 |
|  | (*b*) Describe any methods used to examine subgroups and interactions | N/A |
|  | (*c*) Explain how missing data were addressed | 5 |
|  | (*d*) If applicable, explain how loss to follow-up was addressed | N/A |
|  | (*e*) Describe any sensitivity analyses | 5 |
|  |  |  |
| Participants | (a) Report numbers of individuals at each stage of study—eg numbers potentially eligible, examined for eligibility, confirmed eligible, included in the study, completing follow-up, and analysed | 2 |
|  | (b) Give reasons for non-participation at each stage | 2 |
|  | (c) Consider use of a flow diagram | 2 |
| Descriptive data | (a) Give characteristics of study participants (eg demographic, clinical, social) and information on exposures and potential confounders | 6,16-19 |
|  | (b) Indicate number of participants with missing data for each variable of interest | N/A |
|  | (c) Summarise follow-up time (eg, average and total amount) | N/A |
| Outcome data | Report numbers of outcome events or summary measures over time | 6,16-19 |
| Main results | (*a*) Give unadjusted estimates and, if applicable, confounder-adjusted estimates and their precision (eg, 95% confidence interval). Make clear which confounders were adjusted for and why they were included | 6,16-19 |
|  | (*b*) Report category boundaries when continuous variables were categorized | 4,5 |
|  | (*c*) If relevant, consider translating estimates of relative risk into absolute risk for a meaningful time period | N/A |
| Other analyses | Report other analyses done—eg analyses of subgroups and interactions, and sensitivity analyses | 7 |
| Discussion |  |  |
| Key results | Summarise key results with reference to study objectives | 8 |
| **Limitations** |  |  |
| Interpretation | Give a cautious overall interpretation of results considering objectives, limitations, multiplicity of analyses, results from similar studies, and other relevant evidence | 8,10 |
| Generalisability | Discuss the generalisability (external validity) of the study results | 10 |
| Other information |  |  |
| Funding | Give the source of funding and the role of the funders for the present study and, if applicable, for the original study on which the present article is based | 13 |

****Additional file Table A2.**** Association between preterm birth and HBV-DNA load after multiple imputation.

|  | Total  (N) | Event  (n(%)) | Unadjusted Model | | Model I^*^ | | Model II^†^ | |
| --- | --- | --- | --- | --- | --- | --- | --- | --- |
|  |  |  | crude.OR (95%CI) | crude.*P* | adj.OR (95%CI) | adj.*P* | adj.OR (95%CI) | adj.*P* |
| Group 1 | 559 | 31 (5.55) | 1.00(Ref) |  | 1.00(Ref) |  | 1.00(Ref) |  |
| Group 2 | 339 | 20 (5.90) | 1.07 (0.60-1.91) | 0.824 | 1.06 (0.59-1.89) | 0.857 | 1.11 (0.61-2.03) | 0.728 |
| Group 3 | 163 | 18 (11.04) | 2.11 (1.15-3.89) | 0.016 | 4.90 (2.36-10.15) | < 0.001 | 4.37 (2.05-9.34) | < 0.001 |
| Trend.test |  |  |  | 0.032 |  | 0.001 |  | 0.002 |

^*^Model I adjustment covariates are age, profession, ethnicity, antiviral therapy, and ALT.

^†^Model II adjustment covariates are age, profession, ethnicity, antiviral therapy, ALT, pre-pregnancy BMI, gravidity, parity, residential

status, marital status, in vitro fertilization(IVF), anemia during pregnancy, GDM, history of cesarean delivery, history of preterm birth, and

history of spontaneous abortion.

****Additional file Table A3.** Association between preterm birth and HBV-DNA load after exclusion of women**

**who received antiviral therapy**.

|  | Total  (N) | Event  (n(%)) | Unadjusted Model | | Model I^*^ | | Model II^†^ | |
| --- | --- | --- | --- | --- | --- | --- | --- | --- |
|  |  |  | crude.OR (95%CI) | crude.*P* | adj.OR (95%CI) | adj.*P* | adj.OR (95%CI) | adj.*P* |
| Group 1 | 430 | 26 (6.05) | 1.00(Ref) |  | 1.00(Ref) |  | 1.00(Ref) |  |
| Group 2 | 287 | 17 (5.92) | 0.98 (0.52-1.84) | 0.946 | 0.98 (0.52-1.85) | 0.953 | 0.99 (0.51-1.90) | 0.967 |
| Group 3 | 46 | 12 (26.09) | 5.48 (2.54-11.83) | < 0.001 | 4.77 (2.00-11.37) | < 0.001 | 4.57 (1.84-11.37) | 0.001 |
| Trend.test |  |  |  | 0.002 |  | 0.015 |  | 0.021 |

^*^Model I adjustment covariates are age, profession, ethnicity, antiviral therapy, and ALT.

^†^Model II adjustment covariates are age, profession, ethnicity, antiviral therapy, ALT, pre-pregnancy BMI, gravidity, parity, residential

status, marital status, in vitro fertilization(IVF), anemia during pregnancy, GDM, history of cesarean delivery, history of preterm birth, and

history of spontaneous abortion.
